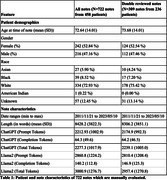# Evaluating Large Language Models (LLMs) in Information Extraction: A Case Study of Extracting Cognitive Exam Dates and Scores

**DOI:** 10.1002/alz.087416

**Published:** 2025-01-09

**Authors:** Hao Zhang, Neil Jethani, Simon Jones, Nicholas Genes, Vincent J Major, Ian S Jaffe, Anthony B Cardillo, Noah Heilenbach, Nadia Fazal Ali, Luke J Bonanni, Andrew Clayburn, Zain Khera, Erica C Sadler, Jaideep Prasad, Jamie Schlacter, Kevin Liu, Benjamin Silva, Sophie Montgomery, Eric J Kim, Jacob Lester, Theodore M Hill, Theodore M Hill, Alba Avoricani, Ethan Chervonski, James Davydov, William Small, Eesha Chakravartty, Himanshu Grover, John Dodson, Abraham A. Brody, Yindalon Aphinyanaphongs, Arjun V. Masurkar, Narges Razavian

**Affiliations:** ^1^ NYU Grossman School of Medicine, New York, NY USA; ^2^ NYU Langone Health, New York, NY USA

## Abstract

**Background:**

Large language models (LLMs) provide powerful natural language processing capabilities in medical and clinical tasks. Evaluating LLM performance is crucial due to potential false results. In this study, we assessed ChatGPT and Llama2, two state‐of‐the‐art LLMs, in extracting information from clinical notes, focusing on cognitive tests, specifically the Mini Mental State Exam (MMSE) and Cognitive Dementia Rating (CDR).

**Method:**

We compiled a dataset consisting of 765 clinical notes mentioning MMSE and CDR. 22 medically trained experts provided the ground truth. ChatGPT (GPT‐4, version “2023‐03‐15‐preview”) and Llama2 (“Llama‐2‐70b‐chat”) were used to extract MMSE and CDR instances with corresponding dates. Inference was successful for 742 notes. We used 20 notes for fine‐tuning and training the reviewers. The remaining 722 were assigned to reviewers, with 309 assigned to two reviewers simultaneously. Precision, sensitivity, true/false negative rates, and accuracy were calculated. For double‐reviewed notes, we qualitatively assessed the errors.

**Result:**

The patient and note characteristics can be found in Table 1. For MMSE information extraction, ChatGPT (vs. Llama2) achieved accuracy of 83% (vs. 66.4%), sensitivity of 89.7% (vs. 69.9%), true‐negative rates of 96% (vs 60.0%), and precision of 82.7% (vs 62.2%). For CDR the results were lower overall, with accuracy of 87.1% (vs. 74.5%), sensitivity of 84.3% (vs. 39.7%), true‐negative rates of 99.8% (98.4%), and precision of 48.3% (vs. 16.1%). We qualitatively evaluated the MMSE errors of ChatGPT and Llama2 on double‐reviewed notes. Llama2 errors included 27 cases of total hallucination, 19 cases where other scores were reported instead of MMSE, 25 missed scores, and 23 cases where the wrong date was reported for the right score. In comparison, ChatGPT’s errors included only 3 cases of total hallucination, 17 cases of wrong test reported instead of MMSE, and 19 cases of reporting a wrong date.

**Conclusion:**

ChatGPT exhibited high accuracy in extracting MMSE scores and dates, with better performance compared to Llama2. The use of LLMs could benefit dementia research and clinical care, by identifying eligible patients for treatments initialization or clinical trial enrollments. Rigorous evaluation of LLMs is crucial to understanding their capabilities and limitations.